# Supplementary material for: Prevalence, Antibiotics Resistance and Plasmid Profiling of Vibrio spp. Isolated from Cultured Shrimp in Peninsular Malaysia
Source: Microorganisms. 2022 Sep 16;10(9):1851. doi: 10.3390/microorganisms10091851 (PMC9505939; doi:10.3390/microorganisms10091851)
Supplement: Supplementary file 1 [file microorganisms-10-01851-s001.zip › microorganisms-1890359-supplementary.pdf]

Supplementary materials

Table S1. Information on *Vibrio* spp. isolates found in this study.

Organism	Strain	Accession No.	Isolation Source	Country
Vibrio communis	S1-3	OP198216	Shrimp: Hepatopancreas	Malaysia: Selangor
Vibrio communis	S1-4	OP198217	Shrimp: Hepatopancreas	Malaysia: Selangor
Vibrio parahaemolyticus	S2-4	OP198218	Shrimp: Hepatopancreas	Malaysia: Selangor
Vibrio sp.	S3-3	OP198219	Shrimp: Hepatopancreas	Malaysia: Selangor
Vibrio sp.	S3-4	OP198220	Shrimp: Hepatopancreas	Malaysia: Selangor
Vibrio sp.	S5-4	OP198221	Shrimp: Hepatopancreas	Malaysia: Selangor
Vibrio sp.	S6-3	OP198222	Shrimp: Hepatopancreas	Malaysia: Selangor
Vibrio campbellii	S7-4	OP198223	Shrimp: Hepatopancreas	Malaysia: Selangor
Vibrio campbellii	S10-3	OP198224	Shrimp: Hepatopancreas	Malaysia: Selangor
Vibrio campbellii	S10-4	OP198225	Shrimp: Hepatopancreas	Malaysia: Selangor
Vibrio parahaemolyticus	S12-3	OP198226	Shrimp: Hepatopancreas	Malaysia: Selangor
Vibrio rotiferianus	S15-3	OP198227	Shrimp: Hepatopancreas	Malaysia: Selangor
Vibrio parahaemolyticus	S15-4	OP198228	Shrimp: Hepatopancreas	Malaysia: Selangor
Vibrio campbellii	S17-3	OP198229	Shrimp: Hepatopancreas	Malaysia: Selangor
Vibrio campbellii	S18-4	OP198230	Shrimp: Hepatopancreas	Malaysia: Selangor
Vibrio parahaemolyticus	S19-3	OP198231	Shrimp: Hepatopancreas	Malaysia: Selangor
Vibrio sp.	S21-3	OP198232	Shrimp: Hepatopancreas	Malaysia: Selangor
Vibrio campbellii	S23-4	OP198233	Shrimp: Hepatopancreas	Malaysia: Selangor
Vibrio rotiferianus	S24-4	OP198234	Shrimp: Hepatopancreas	Malaysia: Selangor
Vibrio sp.	S25	OP198235	Shrimp: Hepatopancreas	Malaysia: Selangor
Vibrio campbellii	S31-3	OP198236	Shrimp: Hepatopancreas	Malaysia: Selangor
Vibrio campbellii	S31-4	OP198237	Shrimp: Hepatopancreas	Malaysia: Selangor
Vibrio sp.	S34-4	OP198238	Shrimp: Hepatopancreas	Malaysia: Selangor
Vibrio sp.	S35-4	OP198239	Shrimp: Hepatopancreas	Malaysia: Selangor
Vibrio parahaemolyticus	M2-4Y	OP198240	Shrimp: Hepatopancreas	Malaysia: Melaka
Vibrio parahaemolyticus	M4-3Y	OP198241	Shrimp: Hepatopancreas	Malaysia: Melaka
Vibrio parahaemolyticus	M4-4G	OP198242	Shrimp: Hepatopancreas	Malaysia: Melaka
Vibrio parahaemolyticus	M4-4Y	OP198243	Shrimp: Hepatopancreas	Malaysia: Melaka
Vibrio parahaemolyticus	M5-3G	OP198244	Shrimp: Hepatopancreas	Malaysia: Melaka
Vibrio rotiferianus	M5-3Y	OP198245	Shrimp: Hepatopancreas	Malaysia: Melaka
Vibrio parahaemolyticus	M6-3	OP198246	Shrimp: Hepatopancreas	Malaysia: Melaka
Vibrio parahaemolyticus	M6-4	OP198247	Shrimp: Hepatopancreas	Malaysia: Melaka
Vibrio parahaemolyticus	M7-3Y	OP198248	Shrimp: Hepatopancreas	Malaysia: Melaka
Vibrio campbellii	M8-4Y	OP198249	Shrimp: Hepatopancreas	Malaysia: Melaka
Vibrio parahaemolyticus	M9-3G	OP198250	Shrimp: Hepatopancreas	Malaysia: Melaka
Vibrio parahaemolyticus	M9-3Y	OP198251	Shrimp: Hepatopancreas	Malaysia: Melaka
Vibrio parahaemolyticus	M9-4G	OP198252	Shrimp: Hepatopancreas	Malaysia: Melaka
Vibrio parahaemolyticus	M9-4Y	OP198253	Shrimp: Hepatopancreas	Malaysia: Melaka
Vibrio parahaemolyticus	M10-3Y	OP198254	Shrimp: Hepatopancreas	Malaysia: Melaka
Vibrio parahaemolyticus	M10-4	OP198255	Shrimp: Hepatopancreas	Malaysia: Melaka
Vibrio parahaemolyticus	M11-3Y	OP198256	Shrimp: Hepatopancreas	Malaysia: Melaka
Vibrio parahaemolyticus	M11-4G	OP198257	Shrimp: Hepatopancreas	Malaysia: Melaka
Vibrio parahaemolyticus	M11-4Y	OP198258	Shrimp: Hepatopancreas	Malaysia: Melaka
Vibrio parahaemolyticus	M12-3G	OP198259	Shrimp: Hepatopancreas	Malaysia: Melaka
Vibrio parahaemolyticus	M12-3Y	OP198260	Shrimp: Hepatopancreas	Malaysia: Melaka
Vibrio parahaemolyticus	J1Y	OP198261	Shrimp: Hepatopancreas	Malaysia: Johor
Vibrio alginolyticus	J3Y1	OP198262	Shrimp: Hepatopancreas	Malaysia: Johor
Vibrio parahaemolyticus	J3Y2	OP198263	Shrimp: Hepatopancreas	Malaysia: Johor
Vibrio parahaemolyticus	J4Y	OP198264	Shrimp: Hepatopancreas	Malaysia: Johor
Vibrio parahaemolyticus	J5Y	OP198265	Shrimp: Hepatopancreas	Malaysia: Johor
Vibrio parahaemolyticus	J6	OP198266	Shrimp: Hepatopancreas	Malaysia: Johor
Vibrio parahaemolyticus	J9Y	OP198267	Shrimp: Hepatopancreas	Malaysia: Johor

Vibrio parahaemolyticus	J10Y	OP198268	Shrimp: Hepatopancreas	Malaysia: Johor
Vibrio parahaemolyticus	J11Y	OP198269	Shrimp: Hepatopancreas	Malaysia: Johor
Vibrio parahaemolyticus	J14Y	OP198270	Shrimp: Hepatopancreas	Malaysia: Johor
Vibrio parahaemolyticus	J15Y	OP198271	Shrimp: Hepatopancreas	Malaysia: Johor
Vibrio parahaemolyticus	J17Y1	OP198272	Shrimp: Hepatopancreas	Malaysia: Johor
Vibrio parahaemolyticus	J17Y2	OP198273	Shrimp: Hepatopancreas	Malaysia: Johor
Vibrio parahaemolyticus	J18Y	OP198274	Shrimp: Hepatopancreas	Malaysia: Johor
Vibrio parahaemolyticus	J21Y	OP198275	Shrimp: Hepatopancreas	Malaysia: Johor
Vibrio parahaemolyticus	J23	OP198276	Shrimp: Hepatopancreas	Malaysia: Johor
Vibrio parahaemolyticus	J24Y1	OP198277	Shrimp: Hepatopancreas	Malaysia: Johor
Vibrio parahaemolyticus	J24Y2	OP198278	Shrimp: Hepatopancreas	Malaysia: Johor
Vibrio parahaemolyticus	J25Y1	OP198279	Shrimp: Hepatopancreas	Malaysia: Johor
Vibrio parahaemolyticus	J25Y2	OP198280	Shrimp: Hepatopancreas	Malaysia: Johor
Vibrio parahaemolyticus	J26Y	OP198281	Shrimp: Hepatopancreas	Malaysia: Johor
Vibrio parahaemolyticus	J29Y1	OP198282	Shrimp: Hepatopancreas	Malaysia: Johor
Vibrio parahaemolyticus	J29Y2	OP198283	Shrimp: Hepatopancreas	Malaysia: Johor
Vibrio parahaemolyticus	J31Y	OP198284	Shrimp: Hepatopancreas	Malaysia: Johor
Vibrio communis	K1Y1	OP198285	Shrimp: Hepatopancreas	Malaysia: Kedah
Vibrio owensii	K3Y2	OP198286	Shrimp: Hepatopancreas	Malaysia: Kedah
Vibrio communis	K4Y1	OP198287	Shrimp: Hepatopancreas	Malaysia: Kedah
Vibrio alginolyticus	K5Y	OP198288	Shrimp: Hepatopancreas	Malaysia: Kedah
Vibrio brasiliensis	K6Y	OP198289	Shrimp: Hepatopancreas	Malaysia: Kedah
Vibrio communis	K7Y	OP198290	Shrimp: Hepatopancreas	Malaysia: Kedah
Vibrio communis	K8Y1	OP198291	Shrimp: Hepatopancreas	Malaysia: Kedah
Vibrio brasiliensis	K8Y2	OP198292	Shrimp: Hepatopancreas	Malaysia: Kedah
Vibrio communis	K9Y	OP198293	Shrimp: Hepatopancreas	Malaysia: Kedah
Vibrio communis	K13Y	OP198294	Shrimp: Hepatopancreas	Malaysia: Kedah
Vibrio communis	K14Y1	OP198295	Shrimp: Hepatopancreas	Malaysia: Kedah
Vibrio owensii	K16Y1	OP198296	Shrimp: Hepatopancreas	Malaysia: Kedah
Vibrio owensii	K16Y2	OP198297	Shrimp: Hepatopancreas	Malaysia: Kedah
Vibrio rotiferianus	K17Y1	OP198298	Shrimp: Hepatopancreas	Malaysia: Kedah
Vibrio rotiferianus	K17Y2	OP198299	Shrimp: Hepatopancreas	Malaysia: Kedah
Vibrio communis	K18Y1	OP198300	Shrimp: Hepatopancreas	Malaysia: Kedah
Vibrio communis	K19Y1	OP198301	Shrimp: Hepatopancreas	Malaysia: Kedah
Vibrio owensii	K21Y	OP198302	Shrimp: Hepatopancreas	Malaysia: Kedah
Vibrio communis	K22Y	OP198303	Shrimp: Hepatopancreas	Malaysia: Kedah
Vibrio brasiliensis	K24Y	OP198304	Shrimp: Hepatopancreas	Malaysia: Kedah
Vibrio rotiferianus	K26Y	OP198305	Shrimp: Hepatopancreas	Malaysia: Kedah
Vibrio alginolyticus	K27Y1	OP198306	Shrimp: Hepatopancreas	Malaysia: Kedah
Vibrio alginolyticus	K27Y2	OP198307	Shrimp: Hepatopancreas	Malaysia: Kedah
Vibrio communis	K28Y1	OP198308	Shrimp: Hepatopancreas	Malaysia: Kedah
Vibrio rotiferianus	K30Y	OP198309	Shrimp: Hepatopancreas	Malaysia: Kedah
Vibrio communis	K33Y2	OP198310	Shrimp: Hepatopancreas	Malaysia: Kedah
Vibrio rotiferianus	K34Y2	OP198311	Shrimp: Hepatopancreas	Malaysia: Kedah
Vibrio communis	K36Y1	OP198312	Shrimp: Hepatopancreas	Malaysia: Kedah
Vibrio alginolyticus	K36Y2	OP198313	Shrimp: Hepatopancreas	Malaysia: Kedah
Vibrio rotiferianus	K37Y1	OP198314	Shrimp: Hepatopancreas	Malaysia: Kedah
Vibrio alginolyticus	K37Y2	OP198315	Shrimp: Hepatopancreas	Malaysia: Kedah
Vibrio owensii	K38Y1	OP198316	Shrimp: Hepatopancreas	Malaysia: Kedah
Vibrio owensii	K38Y2	OP198317	Shrimp: Hepatopancreas	Malaysia: Kedah
Vibrio brasiliensis	K40Y	OP198318	Shrimp: Hepatopancreas	Malaysia: Kedah
Vibrio alginolyticus	K41Y	OP198319	Shrimp: Hepatopancreas	Malaysia: Kedah
Vibrio parahaemolyticus	T1G	OP198320	Shrimp: Hepatopancreas	Malaysia: Terengganu
Vibrio parahaemolyticus	T2G	OP198321	Shrimp: Hepatopancreas	Malaysia: Terengganu
Vibrio parahaemolyticus	T3G	OP198322	Shrimp: Hepatopancreas	Malaysia: Terengganu
Vibrio parahaemolyticus	T4G1	OP198323	Shrimp: Hepatopancreas	Malaysia: Terengganu
Vibrio owensii	T5Y1	OP198324	Shrimp: Hepatopancreas	Malaysia: Terengganu

Vibrio parahaemolyticus	T6G	OP198325	Shrimp: Hepatopancreas	Malaysia: Terengganu
Vibrio parahaemolyticus	T7G1	OP198326	Shrimp: Hepatopancreas	Malaysia: Terengganu
Vibrio parahaemolyticus	T8Y2	OP198327	Shrimp: Hepatopancreas	Malaysia: Terengganu
Vibrio parahaemolyticus	T10Y1	OP198328	Shrimp: Hepatopancreas	Malaysia: Terengganu
Vibrio parahaemolyticus	T11G	OP198329	Shrimp: Hepatopancreas	Malaysia: Terengganu
Vibrio parahaemolyticus	T12Y	OP198330	Shrimp: Hepatopancreas	Malaysia: Terengganu
Vibrio parahaemolyticus	T13G	OP198331	Shrimp: Hepatopancreas	Malaysia: Terengganu
Vibrio parahaemolyticus	T14G	OP198332	Shrimp: Hepatopancreas	Malaysia: Terengganu
Vibrio parahaemolyticus	T15G	OP198333	Shrimp: Hepatopancreas	Malaysia: Terengganu
Vibrio parahaemolyticus	T16G	OP198334	Shrimp: Hepatopancreas	Malaysia: Terengganu
Vibrio owensii	T17G4	OP198335	Shrimp: Hepatopancreas	Malaysia: Terengganu
Vibrio parahaemolyticus	T18G1	OP198336	Shrimp: Hepatopancreas	Malaysia: Terengganu
Vibrio parahaemolyticus	T19G	OP198337	Shrimp: Hepatopancreas	Malaysia: Terengganu
Vibrio owensii	T20G2	OP198338	Shrimp: Hepatopancreas	Malaysia: Terengganu
Vibrio parahaemolyticus	T21Y2	OP198339	Shrimp: Hepatopancreas	Malaysia: Terengganu
Vibrio parahaemolyticus	T22	OP198340	Shrimp: Hepatopancreas	Malaysia: Terengganu
Vibrio owensii	T23G1	OP198341	Shrimp: Hepatopancreas	Malaysia: Terengganu
Vibrio parahaemolyticus	T23G2	OP198342	Shrimp: Hepatopancreas	Malaysia: Terengganu
Vibrio owensii	T23G5	OP198343	Shrimp: Hepatopancreas	Malaysia: Terengganu
Vibrio parahaemolyticus	T24G	OP198344	Shrimp: Hepatopancreas	Malaysia: Terengganu
Vibrio parahaemolyticus	T25G	OP198345	Shrimp: Hepatopancreas	Malaysia: Terengganu
Vibrio parahaemolyticus	T26G	OP198346	Shrimp: Hepatopancreas	Malaysia: Terengganu
Vibrio parahaemolyticus	T28G	OP198347	Shrimp: Hepatopancreas	Malaysia: Terengganu
Vibrio parahaemolyticus	T29G1	OP198348	Shrimp: Hepatopancreas	Malaysia: Terengganu
Vibrio parahaemolyticus	T30G1	OP198349	Shrimp: Hepatopancreas	Malaysia: Terengganu
Vibrio parahaemolyticus	T31G	OP198350	Shrimp: Hepatopancreas	Malaysia: Terengganu
Vibrio parahaemolyticus	T32G	OP198351	Shrimp: Hepatopancreas	Malaysia: Terengganu
Vibrio parahaemolyticus	T33	OP198352	Shrimp: Hepatopancreas	Malaysia: Terengganu
Vibrio owensii	T34Y	OP198353	Shrimp: Hepatopancreas	Malaysia: Terengganu
Vibrio parahaemolyticus	T38G1	OP198354	Shrimp: Hepatopancreas	Malaysia: Terengganu
Vibrio parahaemolyticus	T39G1	OP198355	Shrimp: Hepatopancreas	Malaysia: Terengganu
Vibrio communis	P1Y	OP198356	Shrimp: Hepatopancreas	Malaysia: Perak
Vibrio campbellii	P3Y1	OP198357	Shrimp: Hepatopancreas	Malaysia: Perak
Vibrio rotiferianus	P7G	OP198358	Shrimp: Hepatopancreas	Malaysia: Perak
Vibrio campbellii	P8Y	OP198359	Shrimp: Hepatopancreas	Malaysia: Perak
Vibrio parahaemolyticus	P10G	OP198360	Shrimp: Hepatopancreas	Malaysia: Perak
Vibrio communis	P11Y	OP198361	Shrimp: Hepatopancreas	Malaysia: Perak
Vibrio rotiferianus	P12Y	OP198362	Shrimp: Hepatopancreas	Malaysia: Perak
Vibrio xuii	P13Y1	OP198363	Shrimp: Hepatopancreas	Malaysia: Perak
Vibrio communis	P13Y2	OP198364	Shrimp: Hepatopancreas	Malaysia: Perak
Vibrio parahaemolyticus	P14G	OP198365	Shrimp: Hepatopancreas	Malaysia: Perak
Vibrio harveyi	P19Y	OP198366	Shrimp: Hepatopancreas	Malaysia: Perak
Vibrio rotiferianus	P20Y1	OP198367	Shrimp: Hepatopancreas	Malaysia: Perak
Vibrio campbellii	P21Y	OP198368	Shrimp: Hepatopancreas	Malaysia: Perak
Vibrio parahaemolyticus	P21G	OP198369	Shrimp: Hepatopancreas	Malaysia: Perak
Vibrio parahaemolyticus	P22G	OP198370	Shrimp: Hepatopancreas	Malaysia: Perak
Vibrio harveyi	P23Y1	OP198371	Shrimp: Hepatopancreas	Malaysia: Perak
Vibrio communis	P23Y2	OP198372	Shrimp: Hepatopancreas	Malaysia: Perak
Vibrio campbellii	P24G	OP198373	Shrimp: Hepatopancreas	Malaysia: Perak
Vibrio communis	P25G	OP198374	Shrimp: Hepatopancreas	Malaysia: Perak
Vibrio campbellii	P25Y1	OP198375	Shrimp: Hepatopancreas	Malaysia: Perak
Vibrio campbellii	P25Y2	OP198376	Shrimp: Hepatopancreas	Malaysia: Perak
Vibrio owensii	P27Y	OP198377	Shrimp: Hepatopancreas	Malaysia: Perak
Vibrio parahaemolyticus	P29G	OP198378	Shrimp: Hepatopancreas	Malaysia: Perak
Vibrio campbellii	P28G2	OP198379	Shrimp: Hepatopancreas	Malaysia: Perak
Vibrio xuii	P29Y	OP198380	Shrimp: Hepatopancreas	Malaysia: Perak
Vibrio campbellii	P31Y	OP198381	Shrimp: Hepatopancreas	Malaysia: Perak

Vibrio campbellii	P32Y	OP198382	Shrimp: Hepatopancreas	Malaysia: Perak
Vibrio parahaemolyticus	P33G	OP198383	Shrimp: Hepatopancreas	Malaysia: Perak
Vibrio owensii	P34Y	OP198384	Shrimp: Hepatopancreas	Malaysia: Perak
Vibrio natriegens	V1Y	OP198384	Shrimp: Hepatopancreas	Malaysia: Selangor
Vibrio natriegens	V1Y2	OP198386	Shrimp: Hepatopancreas	Malaysia: Selangor
Vibrio parahaemolyticus	V1G	OP198387	Shrimp: Hepatopancreas	Malaysia: Selangor
Vibrio parahaemolyticus	V1G2	OP198388	Shrimp: Hepatopancreas	Malaysia: Selangor
Vibrio natriegens	V1G2Y	OP198389	Shrimp: Hepatopancreas	Malaysia: Selangor
Vibrio parahaemolyticus	V1G3	OP198390	Shrimp: Hepatopancreas	Malaysia: Selangor
Vibrio natriegens	V2Y	OP198391	Shrimp: Hepatopancreas	Malaysia: Selangor
Vibrio parahaemolyticus	V2G	OP198392	Shrimp: Hepatopancreas	Malaysia: Selangor
Vibrio parahaemolyticus	V3G1	OP198393	Shrimp: Hepatopancreas	Malaysia: Selangor
Vibrio parahaemolyticus	V3G2	OP198394	Shrimp: Hepatopancreas	Malaysia: Selangor
Vibrio parahaemolyticus	V3G3-1	OP198395	Shrimp: Hepatopancreas	Malaysia: Selangor
Vibrio parahaemolyticus	V3Y3	OP198396	Shrimp: Hepatopancreas	Malaysia: Selangor
Vibrio parahaemolyticus	V3Y2G	OP198397	Shrimp: Hepatopancreas	Malaysia: Selangor
Vibrio parahaemolyticus	V3Y3G	OP198398	Shrimp: Hepatopancreas	Malaysia: Selangor
Vibrio parahaemolyticus	V3Y2Y	OP198399	Shrimp: Hepatopancreas	Malaysia: Selangor
Vibrio natriegens	V4Y1	OP198400	Shrimp: Hepatopancreas	Malaysia: Selangor
Vibrio parahaemolyticus	V4G1	OP198401	Shrimp: Hepatopancreas	Malaysia: Selangor
Vibrio parahaemolyticus	V4G2	OP198402	Shrimp: Hepatopancreas	Malaysia: Selangor
Vibrio parahaemolyticus	V4Y3	OP198403	Shrimp: Hepatopancreas	Malaysia: Selangor
Vibrio parahaemolyticus	V5G	OP198404	Shrimp: Hepatopancreas	Malaysia: Selangor
Vibrio parahaemolyticus	V5Y1	OP198405	Shrimp: Hepatopancreas	Malaysia: Selangor
Vibrio parahaemolyticus	V6G	OP198406	Shrimp: Hepatopancreas	Malaysia: Selangor
Vibrio parahaemolyticus	V7Y	OP198407	Shrimp: Hepatopancreas	Malaysia: Selangor
Vibrio parahaemolyticus	V7G1	OP198408	Shrimp: Hepatopancreas	Malaysia: Selangor
Vibrio parahaemolyticus	V7G2	OP198409	Shrimp: Hepatopancreas	Malaysia: Selangor
Vibrio parahaemolyticus	V7G3	OP198410	Shrimp: Hepatopancreas	Malaysia: Selangor
Vibrio parahaemolyticus	V7G3-1	OP198411	Shrimp: Hepatopancreas	Malaysia: Selangor
Vibrio parahaemolyticus	V8G1	OP198412	Shrimp: Hepatopancreas	Malaysia: Selangor
Vibrio parahaemolyticus	V14G2	OP198413	Shrimp: Hepatopancreas	Malaysia: Selangor
Vibrio parahaemolyticus	V14G1	OP198414	Shrimp: Hepatopancreas	Malaysia: Selangor
Vibrio parahaemolyticus	V15G	OP198415	Shrimp: Hepatopancreas	Malaysia: Selangor
Vibrio parahaemolyticus	V22G1	OP198416	Shrimp: Hepatopancreas	Malaysia: Selangor
Vibrio parahaemolyticus	V22Y2	OP198417	Shrimp: Hepatopancreas	Malaysia: Selangor
Vibrio parahaemolyticus	V22G2	OP198418	Shrimp: Hepatopancreas	Malaysia: Selangor
Vibrio parahaemolyticus	V22Y1	OP198419	Shrimp: Hepatopancreas	Malaysia: Selangor
Vibrio parahaemolyticus	V23Y1	OP198420	Shrimp: Hepatopancreas	Malaysia: Selangor
Vibrio parahaemolyticus	V23G1	OP198421	Shrimp: Hepatopancreas	Malaysia: Selangor
Vibrio parahaemolyticus	V23Y2	OP198422	Shrimp: Hepatopancreas	Malaysia: Selangor
Vibrio parahaemolyticus	V24G1	OP198423	Shrimp: Hepatopancreas	Malaysia: Selangor
Vibrio parahaemolyticus	V24G2	OP198424	Shrimp: Hepatopancreas	Malaysia: Selangor
Vibrio parahaemolyticus	V25G	OP198425	Shrimp: Hepatopancreas	Malaysia: Selangor
Vibrio parahaemolyticus	MG2	OP198426	Shrimp: Hepatopancreas	Malaysia: Selangor
Vibrio parahaemolyticus	MG2Y	OP198427	Shrimp: Hepatopancreas	Malaysia: Selangor
Vibrio parahaemolyticus	M2Y2	OP198428	Shrimp: Hepatopancreas	Malaysia: Selangor
Vibrio parahaemolyticus	M5G2	OP198429	Shrimp: Hepatopancreas	Malaysia: Selangor
Vibrio parahaemolyticus	M5Y2	OP198430	Shrimp: Hepatopancreas	Malaysia: Selangor
Vibrio parahaemolyticus	M5Y1	OP198431	Shrimp: Hepatopancreas	Malaysia: Selangor
Vibrio owensii	M6Y	OP198432	Shrimp: Hepatopancreas	Malaysia: Selangor
Vibrio hepatarius	M13Y	OP198433	Shrimp: Hepatopancreas	Malaysia: Selangor
